# Nitric Oxide Destabilizes Pias3 and Regulates Sumoylation

**DOI:** 10.1371/journal.pone.0001085

**Published:** 2007-10-31

**Authors:** Jing Qu, Guang-Hui Liu, Kaiyuan Wu, Peiwei Han, Peng Wang, Jiangmei Li, Xu Zhang, Chang Chen

**Affiliations:** National Laboratory of Biomacromolecules, Institute of Biophysics, Chinese Academy of Sciences, and Graduate School of the Chinese Academy of Sciences, Beijing, China; National Institutes of Health, United States of America

## Abstract

Small ubiquitin-related protein modifiers (SUMO) modification is an important mechanism for posttranslational regulation of protein function. However, it is largely unknown how the sumoylation pathway is regulated. Here, we report that nitric oxide (NO) causes global hyposumoylation in mammalian cells. Both SUMO E2 conjugating enzyme Ubc9 and E3 ligase protein inhibitor of activated STAT3 (Pias3) were targets for S-nitrosation. S-nitrosation did not interfere with the SUMO conjugating activity of Ubc9, but promoted Pias3 degradation by facilitating its interaction with tripartite motif-containing 32 (Trim32), a ubiquitin E3 ligase. On the one hand, NO promoted Trim32-mediated Pias3 ubiquitination. On the other hand, NO enhanced the stimulatory effect of Pias3 on Trim32 autoubiquitination. The residue Cys459 of Pias3 was identified as a target site for S-nitrosation. Mutation of Cys459 abolished the stimulatory effect of NO on the Pias3-Trim32 interaction, indicating a requirement of S-nitrosation at Cys459 for positive regulation of the Pias3-Trim32 interplay. This study reveals a novel crosstalk between S-nitrosation, ubiquitination, and sumoylation, which may be crucial for NO-related physiological and pathological processes.

## Introduction

SUMO modification is an important mechanism for posttranslational regulation of protein function including transcription, intracellular transport, DNA repair, replication, and cell signaling [1]. Three SUMO isoforms (SUMO1, 2, 3) exist. The enzymatic reactions of SUMO modification are similar to those in ubiquitin modification and involve the E1-activating enzyme consisting of an Aos1/Uba2 heterodimer, the E2-conjugating enzyme Ubc9, and the E3 ligase that promotes the transfer of SUMO from the E2 enzyme to substrate proteins. The protein inhibitors of activated STAT (Pias) [2], nuclear pore component RanBP2 [3], and polycomb protein Pc2 [4] have been identified as SUMO E3 ligases. Mammalian cells contain four members of the Pias family (Pias1, 3, x, and y) that share a similar domain structure. These ligases bring the reaction partners in close proximity and this leads to an increased rate of isopeptide bond formation. The conjugation of proteins with SUMO can be reversed by isopeptidases, allowing for dynamic regulation of SUMO-dependent processes [1]. Little is known about the regulation of the sumoylation pathway itself. One example of such regulation is that the viral protein Gam-1 can inhibit sumoylation by targeting E1 and E2 for degradation [5]. In addition, reactive oxygen species (ROS) have been shown to cause repression of sumoylation by crosslinking E1 subunit Uba2 to the E2 subunit Ubc9 [6]. To date, how SUMO E3 ligases are subjected to signal-directed regulation is largely unknown.

Nitric oxide (NO) plays important roles in cell signaling, acting mainly through S-nitrosation of protein cysteine residues [7]. S-nitrosation can link NO signaling to other cellular events through crosstalk with various other post-translational modifications, such as phosphorylation and ubiquitination [7]. However, the connection between S-nitrosation and sumoylation modifications has not been established. We noted that several SUMO-related enzymes contain conserved cysteines, raising the possibility that NO species might influence the biological function of these proteins through direct post-translational modification. In this report, we show that both SUMO E2 (Ubc9) and E3 (Pias) enzymes are targets for S-nitrosation. Importantly, S-nitrosation of Pias3 regulates its stability and thereby counteracts the sumoylation pathway. Our study reveals a novel molecular mechanism through which sumoylation is regulated, which provides new insight into the function of S-nitrosation in human health and disease.

## Results

### Nitric oxide causes global hyposumoylation by a different mechanism than H_2_O_2_

**Figure 1:**
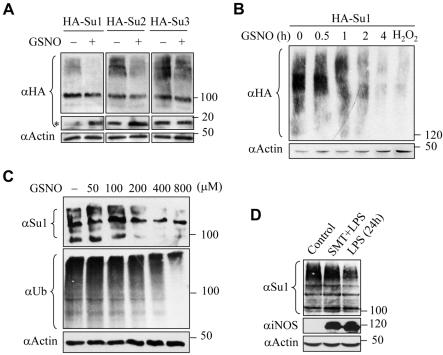
Nitric oxide caused loss of sumo conjugates. (A) HEK293 cells transfected with HA-SUMO1, 2 or 3 were treated with 500 µM GSNO for 4 h, lysed in Laemmli buffer, and immunoblotted with αHA or α actin. Asterisk: free SUMO1/2/3. (B) Time-dependent effect of GSNO on SUMO1 conjugates in transfected HEK293 cells, treated as in (A). In a control experiment, cells were treated with H_2_O_2_ (1 mM) for 1h. (C) HeLa cells were treated with increasing concentrations of GSNO for 4 h, lysed in Laemmli buffer, and immunoblotted with αSUMO1, αubiquitin or αactin. (D) RAW264.7 cells pretreated with or without SMT were stimulated with LPS for 24 h, lysed in Laemmli buffer, and immunoblotted with αSUMO1, αiNOS or αactin.

To investigate whether nitric oxide (NO) can regulate the formation of SUMO-conjugated species, HEK293 cells were transfected with the expression vector encoding the HA-tagged SUMO1, SUMO2, or SUMO3, and then treated with or without 500 µM GSNO (a widly-used NO donor and S-nitrosating agent) for 4 h [7], [8]. The cells were lysed directly in Laemmli buffer and immunoblotted with HA antibody. We observed a marked reduction in the levels of both SUMO1 and SUMO2 conjugates, but only a slight reduction in SUMO3 conjugates (Fig 1*A*). Simultaneous accumulation of free SUMO1 and SUMO2 was also observed in GSNO-challenged cells (Fig 1*A*), suggesting that overall hyposumoylation was attributed to altered utilization rather than reduced expression of SUMO1/2 modifier. Most SUMO1 conjugates disappeared within 4 h, comparable to the effect of 1 h H_2_O_2_ treatment (Fig 1*B*). Notably, several sumoylated substrates, including SP3 and PML, were deconjugated upon GSNO or H_2_O_2_ stimulation (Fig *S1*). The analysis of endogenous SUMO1 conjugates in HeLa cells revealed a dose-dependent repression of overall sumoylation in response to less than 1 mM of GSNO (Fig 1*C*). Correspondingly, global ubiquitination was also downregulated in a dose-dependent manner under GSNO treatment, in sharp contrast to its insensitivity to H_2_O_2_ [6]. It should be pointed out that much higher concentrations (2–8 mM) of GSNO induced a significant increase in overall sumoylation (Fig *S2*), a phenomenon that has recently been reported for high concentrations of H_2_O_2_ [6]. To know whether the decrease in SUMO1 conjugates could occur under conditions of physiological NO production, we employed the RAW264.7 macrophage, which generates NO upon LPS stimulation [9]. Fig 1*D* demonstrates that LPS treatment for 24 h discernibly decreased overall SUMO1 conjugates, accompanied by induction of inducible nitric oxide synthase (iNOS). This effect was prevented by cotreatment with the iNOS inhibitor SMT, indicating that the decrease in SUMO1 conjugates arises from the specific effect of NO.

**Figure 2:**
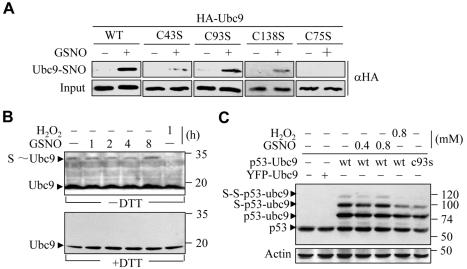
S-nitrosation of Ubc9 did not compromise its SUMO E2 enzyme activity. (A) S-nitrosation of HA-Ubc9 (wild-type or cysteine mutants) in HEK293 cells stimulated with 500 µM GSNO determined by biotin-switch assay. (B) HeLa cells were incubated with 500 µM H_2_O_2_ for 30 min or 500 µM GSNO for the indicated time, lysed in Laemmli buffer with (lower blot) or without (upper blot) DTT, and immunoblotted with αUbc9. (C) HEK293 cells were transfected with the indicated plasmids, treated with H_2_O_2_ (for 1h) or GSNO (for 4 h), lysed in Laemmli buffer, and immunoblotted with αp53 or αactin.

Since H_2_O_2_ results in repression of sumoylation by formation of a disulfide bond between the E1 subunit Uba2 and the E2 Ubc9 [6], we firstly investigated whether NO might involve a similar mechanism. HeLa cells were transfected with HA-Ubc9 and then stimulated with H_2_O_2_ or GSNO. Immunoblotting showed a DTT-sensitive crosslink between Ubc9 and Uba2 upon H_2_O_2_ treatment, consistent with the previous report [6]. GSNO, however, could not induce such a crosslink (Fig *S3*). Considering that Ubc9 contains redox-sensitive cysteines [6], we next investigated whether NO could give rise to Ubc9 thiol modification. HEK293 cells transfected with HA-Ubc9 were treated with GSNO and the lysates were subjected to the biotin-switch assay, in which the S-nitrosation can be specifically identified as described previously [10], [11]. As shown in Fig 2*A*, HEK293-expressed Ubc9 was readily S-nitrosated under GSNO treatment. Using site-directed mutagenesis, we identified Cys75 of Ubc9 as the target for S-nitrosation. Given that the SUMO transfer process involves a *trans*-esterification reaction between Uba2 and Ubc9, we examined whether S-nitrosation of Ubc9 could compromise its ability to receive SUMO. As shown in Fig 2*B*, stimulation of cells with GSNO did not impair the formation of the Ubc9-SUMO thioester bond. In sharp contrast, the thioester intermediate rapidly disappeared upon H_2_O_2_ treatment. Next, in order to know whether S-nitrosation of Ubc9 affects its ability to conjugate SUMO to substrate protein, the Ubc9 fusion-directed sumoylation system, in which Ubc9 can direct sumoylation to fused p53 in a manner independent of E3 ligase [12], was employed. Immunoblotting revealed that the Ubc9-fused p53 was effectively mono- and di-sumoylated in HEK293 cells expressing p53-Ubc9 (Fig 2*C*). The sumoylation of p53 was dependent on the catalytic activity of fused Ubc9, because mutation of Ubc9 at Cys93 diminished such modification and the endogenous p53 was not sumoylated even in the presence of overexpressed Ubc9 (Fig 2*C*), consistent with the previous report [12]. Using this system, we observed that unlike H_2_O_2_, which deconjugated the mono- and di-sumoylated species efficiently, sumoylation of p53 was hardly influenced by GSNO (Fig 2*C*). This experiment indicates that NO does not interfere with the SUMO conjugating activity of Ubc9, provided that Ubc9 and the protein substrate are “close” enough to each other in vivo. To further support the above conclusion that NO does not interfere with the SUMO conjugating function of Ubc9, either Ubc9 (WT) or Ubc9 (C75S) was overexpressed in HEK293 cells (Fig. *S4*). One may predict that, if S-nitrosation of Ubc9 can result in repression of sumoylation, at least overexpression of Ubc9 (C75S), the S-nitrosation-deficient Ubc9 mutant, could prevent GSNO-caused hyposumoylation. However, the outcome was negative (Fig *S4*). Together, these results indicate that NO results in overall hyposumoylation in a Ubc9-independent manner.

### Nitric oxide promotes the degradation of Pias3

**Figure 3:**
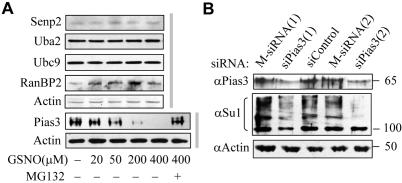
Effects of GSNO on the protein level of SUMO-related enzymes. (A) HeLa cells were treated with increasing concentrations of GSNO for 4 h, lysed in Laemmli buffer and immunoblotted with the indicated antibodies. (B) Immunoblotting analysis of endogenous Pias3 and SUMO1 conjugates in HeLa cells transfected with non-specific, mismatched or Pias3-specific siRNAs.

Since mislocalization of SUMO conjugating or deconjugating enzymes has been reported to result in disruption of the sumoylation pathway [13], [14], we examined the subcellular localizations of SUMO conjugating enzymes (Uba2, Ubc9, and Pias3) and deconjugating enzyme (Senp2), in the absence or presence of GSNO. As shown in Fig *S5*, the intra-nuclear localization of all these enzymes remained unchanged irrespective of the presence of GSNO. Subsequently, we examined their protein expression in HeLa cells. Immunoblotting revealed a dose-dependent decline in the protein level of Pias3, but not Uba2, Ubc9 and Senp2 under GSNO stimulation (Fig 3*A*). In a control experiment, the expression of RanBP2 slightly increased under the same conditions, thereby indicating a specific down-regulation of Pias3 among the SUMO E3 ligases. Preincubation of cells with proteasome inhibitor MG132 prevented the disappearance of Pias3 caused by GSNO (Fig 3*A*), suggesting that NO might regulate Pias3 degradation in a proteasome-dependent manner. To evaluate whether the reduced level of Pias3 could account for global hyposumoylation, two different small interfering RNAs (siRNA) specific to Pias3 were transfected into HeLa cells. As shown in Fig 3*B*, knockdown of endogenous Pias3 substantially reduced most SUMO1 conjugates, indicating that the degradation of endogenous Pias3 was responsible for NO-elicited repression of sumoylation, although other mechanisms may also be involved.

### Nitric oxide promotes Pias3-Trim32 interplay and enhances Pias3 ubiquitination

**Figure 4:**
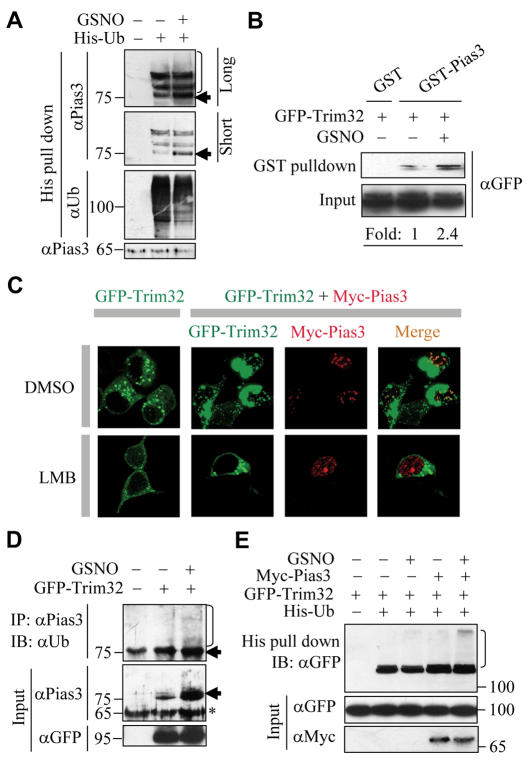
GSNO promotes Pias3-Trim32 interplay. (A) HeLa cells expressing His-Ub were stimulated with or without GSNO for 4 h and then subjected to His-Ub pull down. Both longer and shorter exposures are shown. Bracket: poly-ubiquitinated Pias3; Arrow: mono-ubiquitinated Pias3. (B) Effect of GSNO on Pias3-Trim32 interaction determined by GST-pull down assay. GST-Pias3 fusion proteins, immobilized on beads, were mixed with cell lysates with GFP-Trim32 expression in the absence or presence of 500 µM GSNO. Pull-downed and input GFP-Trim32 was analyzed by immunoblotting. (C) Confocal microscopic analysis of subcellular localization of Myc-Pias3 and GFP-Trim32 in cotransfected HEK293 cells. 4 h after transfection, cells were incubated with or without LMB for 16 h. (D) HeLa cells transfected with or without GFP-Trim32 were stimulated with or without GSNO for 4 h and then the denatured lysates were immunoprecipitated with αPias3, followed by detection for Ub. Bracket: poly-ubiquitinated Pias3; Arrow: mono-ubiquitinated Pias3; Asterisk: unmodified Pias3. (E) HEK293 cells transfected with the indicated plasmids were stimulated with or without GSNO for 1 h and then subjected to His-Ub pull down. Bracket: ubiquitinated GFP-Trim32. In panels (A), (C), (D) and (E), cells were incubated with MG132 to prevent potential protein degradation.

Proteins are usually ubiquitinated before proteasomal degradation [15], [16]. To find out whether NO promotes ubiquitination of Pias3, HeLa cells were transfected with His-tagged ubiquitin, and the ubiquitin-conjugated proteins were recovered with Ni^2+^ affinity agrose and subjected to immunoblotting for Pias3. As shown in Fig 4*A*, the ubiquitinated forms of Pias3 (especially the mono-ubiquitinated one) obviously increased after cells were treated with GSNO. This increase was specific, because the overall amount of ubiquitin conjugates diminished under the same conditions.

The observation that NO selectively promoted Pias3 ubiquitination suggested a possibility that NO might enhance the specific interaction between Pias3 and its ubiquitin E3 ligase. Trim32, a RING-containing protein, has recently been reported to promote ubiquitination of Piasy [15]. To assess the possibility that Pias3 employs Trim32 as its ubiquitin E3 ligase, we firstly utilized a GST pull-down assay to explore the potential association between Pias3 and Trim32. The results demonstrated that the recombinant GST-Pias3, but not GST alone, specifically associated with Trim32 (Fig 4*B*). Such association was also verified in intact cells by co-immunoprecipitation (Co-IP) (Fig *S6* and Fig *S7*). Subsequently, it was investigated whether Pias3 and Trim32 colocalize in the cells. HEK293 cells were cotransfected with GFP-Trim32 and Myc-Pias3 and then their subcellular localizations were determined. As shown in Fig 4*C*, Trim32 mainly resided in cytoplasmic small granules in Trim32-singly expressed cells. Surprisingly, coexpression of Pias3 induced the appearance of Trim32 in Pias3-localized nuclear dots. In addition, Trim32 was found in large cytosolic aggregates upon Pias3 cotransfection (Fig 4*C* and Movie *S1*), a phenomenon that may be explained by the finding that Pias3 stimulated autoubiquitination of Trim32 (Fig 4*E*). To determine the mechanism by which Trim32 was recruited to the Pias3-localized nuclear compartment, LMB, a CRM1-targeting nuclear export inhibitor, was used. As shown in Fig 4*C*, LMB prevented the colocalization of Trim32 and Pias3 in the nucleus as well as preventing the stimulatory effect of Pias3 on Trim32 cytosolic aggregation. Considering that Pias proteins bear both a nuclear localization sequence and a nuclear export sequence [15], we speculated that the import of Trim32 into the nuclear dots might be mediated by the dynamic nucleocytosolic shuttling of Pias3.

To investigate whether Trim32 controls ubiquitination of Pias3, HeLa cells were transiently transfected with GFP-Trim32, and then the endogenous Pias3 was immumoprecipitated from the denatured cell lysates, followed by immunoblotting analysis for ubiquitin. As shown in Fig 4*D*, both poly- and mono-ubiquitinated Pias3 were elevated in the presence of overexpressed Trim32. It should be pointed out that although mono-ubiquitination may not necessarily be required for regulation of protein stability, degradation of either actin or Piasy by Trim32 has been shown to correlate with mono-ubiquitination [15], [17]. Next, we explored the possible effect of Pias3 on Trim32, including autoubiquitination and potential sumoylation of Trim32. It turned out that Trim32 could not be sumoylated, even in the presence of overexpressed Pias3 (data not shown). Interestingly, the autoubiquitination of Trim32, an indicator of ubiquitin E3 ligase activity [10], [15], was enhanced by cotransfected Pias3 (Fig 4*E*), indicating that association with Pias3 elevates the ligase activity of Trim32. Finally, we examined the possible regulatory role of NO on the Trim32-Pias3 interplay. It was observed in a GST pulldown assay that addition of GSNO markedly strengthened the Pias3-Trim32 association (Fig 4*B*). A Co-IP experiment demonstrated that the affinity between Pias3 and Trim32 was substantially increased in HEK293 cells ectopicly expressing iNOS (Fig *S7*), indicating that the increased association occurs in cells where endogenous NO is generated. As for the functional interplay between Pias3 and Trim32, both Trim32-mediated Pias3 ubiquitination and Pias3-stimulated autoubiquitination of Trim32 were found to be substantially enhanced in the presence of GSNO (Fig 4*D, E*). Together, these observations indicate that NO promotes the association and bidirectional influence between Trim32 and Pias3, which may facilitate Trim32-mediated Pias3 ubiquitination.

### S-nitrosation of Pias3 positively regulates Pias3-Trim32 association

**Figure 5:**
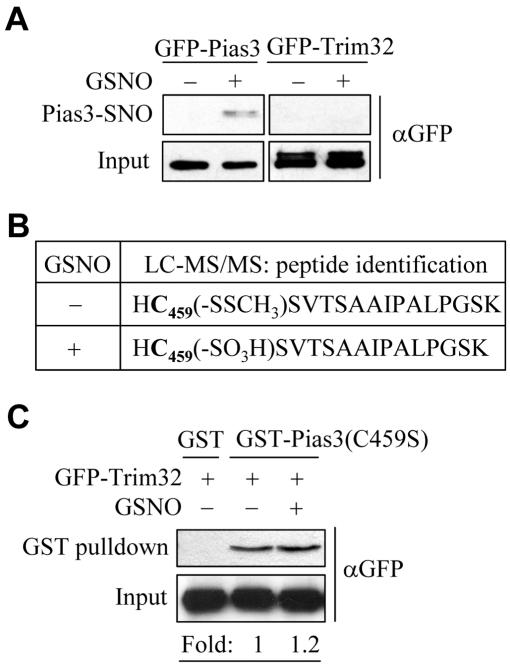
S-nitrosation of Pias3 and its effects on Pias3-Trim32 association. (A) S-nitrosation of GFP-Pias3 in HEK293 cells stimulated with 500 µM GSNO determined by biotin-switch assay. (B) Recombinant Pias3 was exposed to 100 µM GSNO and blocked with MMTS. In-gel digested Pias3 was subjected to Nano-LC-MS/MS analysis. (C) Effect of GSNO on Pias3(C459S)-Trim32 interaction determined by GST-pull down assay.

Since S-nitrosation has been reported to facilitate protein-protein interactions [9], we speculated that NO-mediated specific molecular modification of Pias3 (or Trim32) might regulate its interaction with Trim32 (or Pias3). Using the biotin-switch method, we verified that Pias3, as well as other members of the Pias family, could be efficiently S-nitrosated (Fig 5*A* and Fig *S8*). The absence of S-nitrosated Trim32 under GSNO treatment revealed the specificity of the above modifications (Fig 5*A*). Furthermore, Pias3 was found to be constitutively S-nitrosated in HEK293 cells ectopicly expressing iNOS (Fig *S9*), indicating a role of endogenously produced NO in modifying Pias3. To locate the S-nitrosated cysteine residue(s) of Pias3, recombinant Pias3 protein was exposed to GSNO, blocked with MMTS, and then subjected to mass spectrometry analysis. As shown in Fig 5*B* and Fig *S10*, the Cys459 of Pias3 was oxidized to sulphonic acid (-SO_3_H) only after exposure to GSNO. Considering that S-nitrosation can facilitate further oxidation of the same cysteine thiol [9], [18], and Cys459 is located within a hydrophobic pocket (Fig *S9*), a factor known to govern S-nitrosation specificity [19], this result implies that Cys459 is a S-nitrosation site. To further confirm this, HEK293-expressed Pias3 mutant (C459S), whose cysteine 459 was mutated to serine, was subjected to the biotin-switch assay. As expected, mutation of Pias3 at Cys459 substantially diminished its S-nitrosation (Fig *S11*). Next, we investigated whether S-nitrosation of Pias3 regulated its association with Trim32. It was demonstrated by GST-pull down assay that although the Pias3 bearing a C459S mutation still retained the ability to associate with Trim32 in the absence of GSNO, the stimulatory effect of GSNO on Pias3(C459S)-Trim32 association disappeared (Fig 5*C*). In addition, the GSNO-enhanced binding of wild-type Pias3 to Trim32 could be abolished by dithiothreitol (DTT), a nitrosothiol-reducing reagent (data not shown). Together, these results indicate that S-nitrosation of Pias3 at Cys459 is required for positive tuning of the Pias3-Trim32 association.

## Discussion

**Figure 6:**
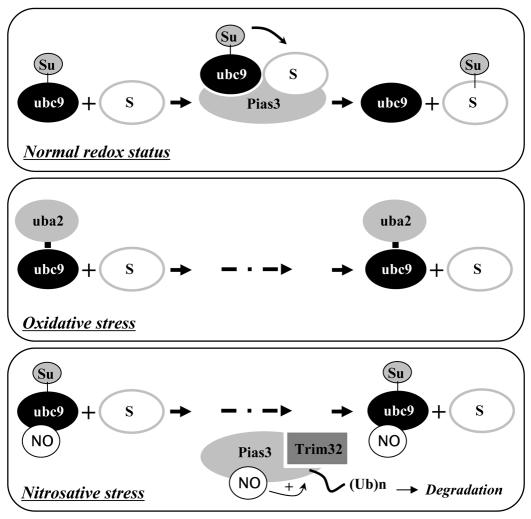
Model demonstrating that oxidative and nitrosative stress block the sumoylation pathway through different mechanisms. Top: In a normal redox environment, Ubc9 conjugates SUMO to the substrate with the help of E3 ligase. Middle: Oxidative stress leads to formation of a disulfide bond between the E1 subunit Uba2 and the E2 subunit Ubc9, resulting in inactivation of both E1 and E2 enzymes. Below: Under nitrosative stress, both Ubc9 and Pias3 are S-nitrosated. Whereas S-nitrosation of Ubc9 cannot interfere with its catalytic activity, S-nitrosation of Pias3 facilitates its degradation by promoting its interplay with Ub E3 ligase Trim32, thereby resulting in decrease of SUMO conjugating efficiency. Su, SUMO; S, substrate protein.

Post-translational protein modification is a crucial mechanism for regulation of protein function in eukaryotic cells. S-nitrosation has gained increasing recognition as a functionally important post-translational modification, which exerts pleiotropic effects on its target proteins ranging from altered protein stabilization to changed activity or function [7]. Here we describe the S-nitrosation of Pias3 and show that this modification enhances its association with Trim32, a ubiquitin E3 ligase, which results in Pias3 degradation and repression of sumoylation (Fig 6). This work may have implications in three different conceptual areas. First, it reveals a unique mechanism to counteract the sumoylation pathway. Second, it provides new evidence that S-nitrosation regulates protein-protein interactions. Third, it reveals an unexpected crosstalk between S-nitrosation, sumoylation and ubiquitination.

Firstly, we demonstrated a novel biological phenomenon, namely that NO led to desumoylation of most SUMO targets, which was due to at least in part, destabilization of SUMO E3 ligase Pias3. Unlike the effects of low concentrations of H_2_O_2 _or the virus protein Gam1, both of which target SUMO E1 and E2 enzymes, NO failed to interfere with Ubc9-mediated transfer of SUMO from Uba2 to the target protein, although it could S-nitrosate Ubc9 at a non-catalytic cysteine. Despite targeting E3, the efficiency of NO in the repression of global sumoylation is high. The effect obtained with 500 µM GSNO was comparable to that of 1 mM H_2_O_2_, a much stronger oxidant. Moreover, the endogenous NO produced in LPS-stimulated macrophages demonstrated a slight inhibitory effect on overall sumoylation, an effect that is however more significant than that obtained with endogenous H_2_O_2_ from activated NADPH oxidase [6]. It should be pointed out that, although S-nitrosation-dependent degradation of Pias3 contributes to loss of SUMO conjugates (Fig.3*B*), global sumoylation profile may be controlled by the net effect of NO on multiple protein targets. For example, Pias family members including Pias3, Piasxα, Piasxβ and Piasy were all found as S-nitrosated targets (Fig *S8*). In addition, most of SUMO isopeptidases (such as Senp) contain redox-sensitive cysteine(s). If these isopeptidases can be inhibited by higher concentrations of NO, one may understand why high concentration of GSNO showed a stimulatory effect on overall sumoylation [6]. In fact, it is not contradictory that various SUMO-related enzymes coordinately respond to certain signal and control sumoylation. This is indeed evidenced by a recent report that the cAMP pathway repressed global sumoylation in human endometrial stromal cells by differentially affecting the protein contents of various SUMO E3 ligases and isopeptidases [20].

Concerning the molecular mechanism by which NO promotes Pias3 degradation, we observed the S-nitrosation-stimulated recruitment of ubiquitin E3 ligase to Pias3. This biological phenomenon reflects S-nitrosation-tuned protein-protein interactions. In our study, S-nitrosation of a single cysteine C459 was found to be sufficient to mediate regulation of association of Pias3 and Trim32. However, C459 is not located in the N-terminal region of Pias3 that harbors the Trim32-binding site (data not shown), suggesting that S-nitrosation probably regulates Pias3-Trim32 interaction by inducing a conformational change in Pias3. At this point, our finding seems similar to a report that S-nitrosation of GAPDH at a single cysteine stimulates its association with Siah1, a ubiquitin E3 ligase [9]. In the present study, NO enhanced Pias3-Trim32 affinity but did not affect the intranuclear localization of Pias3, suggesting that ubiquitination of Pias3 might occur in the nucleus. During preparation of this manuscript, Depaux et al reported an intranuclear degradation of Pias proteins by Siah2, another ubiquitin E3 ligase [16]. In cells coexpressing Pias1 and Siah2, Siah2 was translocated from the cytosol to the nucleus where it colocalized with Pias1, and was distributed into cytosolic aggregates [16]. This report is highly consistent with our finding, and suggests a possibility that NO may also stimulate the interplay of Pias3 with other ubiquitin E3 ligases. Unraveling the effect of NO in the regulation of the Siah2-Pias pathway would be an interesting topic for future study.

Although SUMO and ubiquitin pathways are highly similar, little is known about signal-directed coupling of the two systems. We show here that NO links ubiquitination to sumoylation by promoting the binding of ubiquitin and SUMO E3 ligases, a process intergrating three kinds of post-translational modifications: S-nitrosation, sumoylation and ubiquitination. Recently, a crosstalk between S-nitrosation and ubiquitination has been reported [10]. NO was shown to inhibit the ubiquitin E3 ligase activity of Parkin through S-nitrosation [10]. By such mechanism, NO might cause disappearance of the bulk of ubiquitin conjugates, as observed in Fig 1*C*. In fact, based on a similar idea, we have investigated the possible influence of S-nitrosation on the SUMO E3 ligase activity of Pias3; however, the result was negative (data not shown). The insensitivity of Pias3 activity to NO is consistent with the fact that the C459 of Pias3 that is S-nitrosated does not reside in the catalytic RING domain. Therefore, we speculate that S-nitrosation probably serves mainly as a negative regulatory mechanism to control the stability of Pias3 and thereby tune the sumoylation pathway. However, it should not be ignored that Pias3 protein itself is a negative regulator of pro-inflammatory gene transcription [21], [22]. Thus, Pias3 may also serve as an intranuclear NO sensor, whose degradation represents a step to turn on inflammatory gene expression under nitrosative stress.

In summary, we report here that NO regulates sumoylation through S-nitrosation-dependent degradation of Pias3. Since SUMO modification participates in the regulation of multiple protein functions, repression of sumoylation may mediate a number of NO-related cellular events and pathological processes. For instance, desumoylation of PML and SP3 may be involved in NO-elicited transcriptional regulation. In addition, NO has been shown to desumoylate DJ-1 in neural cells, whose sumoylation is required for the full protective functions of DJ-1 (unpublished observation). Therefore, identifying proteins whose SUMO modification is reduced under nitrosative stress and unraveling the related functional consequences presents an exciting and important challenge for future research.

## Materials and Methods

### Reagents, plasmids and antibodies

Lipopolysaccharide (LPS), S-methyl thiocarbamate (SMT), N-ethylmaleimide (NEM), iodoacetamide, and MG132 were purchased from Sigma. Methyl methanethionsulphonate (MMTS) and *N*-[6-(biotinamido)hexyl]-3′-(2′-pyridyldithio) propionamide (Biotin-HPDP) were purchased from Pierce. Other reagents have previously been described [8]. The following expression plasmids were described in previous investigations: HA-SUMO1(GG), SUMO2(GG), and SUMO3(GG) [23], HA-Ubc9 [24], p53-Ubc9 [12], Flag-Uba2 [6], GFP-SENP2 [13], GFP-Pias3 [25], YFP-Ubc9 [4], Myc-Pias3 [26], GFP-Trim32 [17], iNOS [27], HA-Pias3, and GST-Pias3 [28]. Mutants of Ubc9 and Pias3 were engineered by site-directed mutagenesis (Stratagene). Myc, HA, Flag, His, Ub, p53, Pias3, SUMO1, GFP monoclonal antibodies and Ubc9, Pias3, RanBP2, Senp2, Trim32, GFP, actin polyclonal antibodies were purchased from Santa Cruz. SUMO1 and Uba2 monoclonal antibodies were purchased from Zymed and BD Pharmingin, respectively. iNOS polyclonal antibody was from BD Pharmingin.

### Cell transfection and immunoblotting

HeLa and HEK293 cells were respectively transfected with lipofectamine 2000 (Invitrogen) and JetPei (Polyplus) according to the manufacturer's protocol. For sumoylation analysis, cells were lysed directly in Laemmli buffer supplemented with 20 mM NEM and iodoacetamide. Extracts were boiled for 30 min prior to loading on SDS-polyacrylamide gels. Immunoblotting analysis was performed as previously described [29].

### Ubiquitination analysis

Cells were transfected with His-Ub together with other expression plasmids. 36 h after transfection, cells were incubated with MG132 and then lysed in 1 ml of lysis buffer (6 M guanidinium HCl, 0.1 M Na_2_HPO_4_/NaH_2_PO_4_, and 10 mM Tris-HCl, pH 8.0). After sonication and centrifugation, 90% of the lysate was incubated with 25 µl of Ni^2+^ Sepharose beads (Amersham) for 3 h. The beads were washed twice with lysis buffer, followed by washing three times with washing buffer (8 M urea, 0.1 M Na_2_HPO_4_/NaH_2_PO_4, _pH 6.4). After a final wash with phosphate-buffered saline, the beads were treated in SDS sample buffer for SDS-PAGE. 1/10th of the cells were lysed in RIPA and the lysates were used as input controls.

### RNA interference

The non-targeting siRNA (siControl) we used was UUCUCCGAACGUGUCACGUTT. Two different siRNAs targeting human Pias3 were UUGGUCAUCUGAGUUCGGAdTdT (siPias3-1) [28] and GGAGCCAAAUGUGAUUAUAdTdT (siPias3-2) [30]. To evaluate the specificity of the above siRNA, the mismatched siRNAs (M-siRNA) with only a three-base difference were employed. M-siRNAs for siPIAS3(1) and siPIAS3(2) were UUGGUCAUAGUAGUUCGGAdTdT and GGAGCCAACGUUGAUUAUAdTdT respectively. The siRNAs were transfected into HeLa cells using Lipofectamine 2000 according to the manufacturer's protocol. After 36 h, the cells were transfected again for another 36 h. The protein level of Pias3 was then monitored by immunoblotting.

### Immunofluorescence analysis

HEK293 cells were transfected with Myc-Pias3 and GFP-Trim32 expression vectors. 4 h later, cells were incubated with or without LMB for 16 h. Immunofluorescence analysis was performed with αMyc primary antibody and Texas Red-conjugated secondary antibody, as previously described [8]. Pictures of cells mounted in ProLong Antifade (Molecular Probes) were taken with a laser-scanning confocal microscope (Olympus FV500, Tokyo, Japan), 60x PlanApo lens (aperture 1.4, oil), using FLUOVIEW software.

### Immunoprecipitations

Cells were lysed in ice-cold lysis buffer (20 mM Tris-HCl (pH 7.5), 150 mM NaCl, 10 mM NaF, 1 mM EDTA, 10% glycerol, 0.1% NP-40, 10 µM MG132, and the protease inhibitor cocktail). For immunoprecipitation using harsh denaturing conditions (Ub assay), the final concentration of sodium dodecyl sulphate (SDS) in the lysis buffer was increased to 2% and after boiling for 5 min, lysates were diluted into lysis buffer lacking SDS to give a final concentration of 0.1% SDS. Corresponding agarose bead-conjugated antibodies were incubated with 500 µg cell lysate, and the immunoprecipitates were analyzed by immunoblotting with appropriate antibodies.

### GST pull-down assay

GFP-Trim32 protein, obtained from the whole cell lysate of transfected HEK293 cells, was mixed with GST or GST-Pias3 bound to Sepharose beads in 1ml of binding buffer (20 mM Tris-HCl (pH 7.5), 150 mM NaCl, 1 mM EDTA, 10% glycerol, 0.1% NP-40, 10 µM MG132, and the protease inhibitor cocktail). 500 µM GSNO was then added to the mixture and incubated with rotation for 2 h at room temperature in dark. The beads were washed three times with 1 ml binding buffer, separated by SDS–PAGE and analyzed by immunoblotting.

### Biotin-switch assay

Cell lysates were prepared in lysis buffers (50 mM Tris (pH 7.5), 150 mM NaCl, 0.5% deoxycholic acid, 0.1% SDS, 1% NP-40, 0.1 mM neocuproine, and the protease inhibitor cocktail). The supernatant of cell lysis was analyzed for protein concentration by the BCA method and then adjusted to the appropriate concentration. Blocking buffer (2.5% SDS, 20 mM MMTS in HEN buffer) was mixed with the samples and incubated for 30 min at 50°C to block free thiol groups. After removing excess MMTS by acetone precipitation, nitrosothiols were then reduced to thiols and biotinylated by reducing buffer (1% SDS, 10 mM ascorbate, 4 mM Biotin-HPDP). The biotinylated proteins were then pulled down by streptavidin-agarose beads, eluted by SDS sample buffer and subjected to immunoblotting.

### Pias3 site identification by (nano) Liquid Chromatography-Mass Spectrometry

Mass spectra were acquired after in-gel tryptic digestion of recombinant Pias3 on a Thermo LTQ linear trap instrument equipped with a Thermo nano electrospray source and a Thermo Surveyor pump and autosampler (Thermo Electron Corporation, San Jose, CA) according to a previously described method with minor modifications [9], [18]. Briefly, recombinant Pias3 was exposed to 100 µM of GSNO *in vitro* for 30 min at 37°C. Remaining free thiols in Pias3 were blocked with 20 mM methyl methanethiosulphonate (MMTS) in blocking buffer (25 mM Hepes, pH 7.7, 1 mM EDTA, 0.1 mM neocuproine, 2.5% SDS) at 50°C for 30 min with frequent vortexing followed by separation by non-reducing SDS-PAGE in the dark. The gel was stained with Commassie bright blue, cut and subjected to in-gel tryptic digestion. LC-MS/MS analyses were performed on a fused silica capillary (75 µm i.d.×12 cm) packed with Synergi Hydro-RP silica (reverse-phase, 4 μ, 80 Å, Phenomenex). Mobile phase A consisted of 5% acetonitrile/ 0.1% formic acid and mobile phase B was 80% acetonitrile/ 0.1% formic acid. Peptides were eluted initially with 100% A for 1 min, then 90% A for 5 min, then a linear gradient to 55% A by 60 min, then to 0% A at 70 min and held to 80 min, then to 100% A at 80.01 min and held until 90 min. MS/MS spectra were acquired by using a full scan followed by five MS/MS scans in a data-dependent mode. Precursors that were detected twice within 15 s were put on a dynamic exclusion list for a period of 60 s. MS/MS data were processed with in-house Bioworks (version 3.2, Thermo Electron Corporation, San Jose, CA) as described below: MS/MS spectra were extracted from the raw files by using SEQUEST with the following parameters; MW range: 250–4,000; threshold, 1,000; precursor mass tolerance: 2; group scan tolerance, 2; minimum group count, 1; precursor charge state, auto; MSn level, auto. Raw data were searched against NCBI human Refseq protein database (version: 2006.9.18) and filtered with the following criteria: Xcorr>1.9 for singly charged, >2.5 for doubly charged and >3.75 for triply charged ions; Delta Cn>0.1; RSp<5; and preliminary score (Sp)>500. Cysteines either modified by MMTS (+46), nitrosation (+29), sulphination (+32) or sulphonation (+48) and methionine oxidation (+16) were all specified as differential modifications. MS/MS spectra were manually validated by the following criteria: (i) a continuous b or y-ion series of at least five residues and (ii) the top three most intense fragment peaks assigned to either an a, b, y-ion, to an a, b, y-ion resulting from a neutral loss of water or ammonia, or to a multiply protonated fragment ion. All acquired data were further validated by Trans-Proteomics Pipeline (TPP, Institute of System Biology, Seattle) with PeptideProphet®(Keller et al., 2002) >0.99 and ProteinProphet® = 1.0 as a threshold for peptides and protein validation, respectively (data not shown). The above results represent three independent experiments.

Materials used: MMTS (Pierce), sequencing-grade trypsin (Promega), formic acid and acetonitrile were HPLC-grade from J.T. Baker Chemicals. Synergi Hydro-RP silica (4 μ, 80 Å) was from Phenomenex.

## Supporting Information

Figure S1 HEK293 cells transfected with GFP-PML (left) or HA-SP3 (right) were treated with H2O2 (1 mM) for 1h or with GSNO (0.5 mM) for indicated time, lysed in Laemmli buffer and immunoblotted with anti-GFP or anti-HA.
(0.29 MB TIF)

Figure S2 HeLa cells were treated with increasing concentrations of GSNO for 4 h, lysed in Laemmli buffer, and immunoblotted with anti-SUMO1 or anti-actin.
(0.09 MB TIF)

Figure S3 HeLa cells transfected with HA-Ubc9 were incubated with 0.5 mM H2O2 for 30 min or with 0.5 mM GSNO for the indicated time, lysed in Laemmli Buffer with (lanes 3 and 10) or without (other lanes) DTT and immunoblotted with either anti-HA (left) or anti-Uba2 (right). Asterisk: unspecific crossreacting band.
(0.21 MB TIF)

Figure S4 HeLa cells were cotransfected with His-SUMO1 and increasing amounts (from 30 to 300ng) of HA-Ubc9(WT) or HA-Ubc9(C75S), treated with 0.5 mM GSNO for 4 h, lysed in Laemmli buffer, and immunoblotted with anti-His.
(0.39 MB TIF)

Figure S5 HEK293 cells were transfected with the indicated plasmids and subjected to immunofluorescent (for Flag-Uba2) or fluorescent protein imaging.
(0.26 MB TIF)

Figure S6 Co-IP analysis of the Pias3-Trim32 interaction in MG132-treated HeLa cells using anti-Trim32 (or control IgG) as the IP antibody. No coimmunoprecipitation was found between Trim32 and RanBP2, indicating a specific interaction between Trim32 and Pias3.
(0.07 MB TIF)

Figure S7 Co-IP analysis of Pias3-Trim32 interaction in transfected HEK293 cells. HEK293 cells were cotransfected with the indicated plasmids. 36h later, cells were treated with MG132 for 4h. The lysates were subjected to CO-IP assay using anti-Myc as the IP antibody. The expression of Myc-Pias3, GFP-Trim32 and/or iNOS in immunoprecipitates and cell lysates were monitored with the indicated antibodies.
(0.11 MB TIF)

Figure S8 S-nitrosation of various Flag-tagged Pias subtypes expressed in HEK293 cells in the presence of 0.5 mM GSNO was determined by biotin-switch assay.
(0.13 MB TIF)

Figure S9 S-Nitrosation of HA-Pias3 in iNOS-overexpressed HEK293 cells.
(0.05 MB TIF)

Figure S10 Nano-LC-MS/MS analysis of Pias3 before and after 0.1 mM GSNO treatment indicating cysteine-459 (C459) on peptide 458HCSVTSAAIPALPGSK473 was the modified site of S-nitrosation. The m/z 795.22+ precursor ion corresponds to the Pias3 peptide 458HCSVTSAAIPALPGSK473 with cysteine sulphonation in the GSNO-treated sample. Cysteine sulphonation was only observed after exposure to the NO donor GSNO. Analysis of the informative fragment ions y14+, y15+ confirmed the presence of the sulphonated cysteine. Kyte-Doolittle hydropathic index plot from the region flanking the identified S-nitrosocysteine residue (arrow) also showed that Cys459 was located within a hydrophobic pocket. The hydropathy plot was constructed by using a window of 13 amino acids.
(1.73 MB TIF)

Figure S11 S-nitrosation of HA-Ubc9 or its C459S expressed in HEK293 cells in the presence of 0.5 mM GSNO was determined by biotin-switch assay.
(0.11 MB TIF)

Movie S1 3D presentation of Trim32-Pias3 colocalization in the nucleus
(10.30 MB AVI)

## References

[1] Hay RT (2005). SUMO: a history of modification.. Mol Cell.

[2] Kotaja N, Karvonen U, Janne OA, Palvimo JJ (2002). PIAS proteins modulate transcription factors by functioning as SUMO-1 ligases.. Mol Cell Biol.

[3] Pichler A, Gast A, Seeler JS, Dejean A, Melchior F (2002). The nucleoporin RanBP2 has SUMO1 E3 ligase activity.. Cell.

[4] Kagey MH, Melhuish TA, Wotton D (2003). The polycomb protein Pc2 is a SUMO E3.. Cell.

[5] Boggio R, Colombo R, Hay RT, Draetta GF, Chiocca S (2004). A mechanism for inhibiting the SUMO pathway.. Mol Cell.

[6] Bossis G, Melchior F (2006). Regulation of SUMOylation by reversible oxidation of SUMO conjugating enzymes.. Mol Cell.

[7] Hess DT, Matsumoto A, Kim SO, Marshall HE, Stamler JS (2005). Protein S-nitrosylation: purview and parameters.. Nat Rev Mol Cell Biol.

[8] Qu J, Liu GH, Huang B, Chen C (2007). Nitric oxide controls nuclear export of APE1/Ref-1 through S-nitrosation of Cysteines 93 and 310.. Nucleic Acids Res.

[9] Hara MR, Agrawal N, Kim SF, Cascio MB, Fujimuro M (2005). S-nitrosylated GAPDH initiates apoptotic cell death by nuclear translocation following Siah1 binding.. Nat Cell Biol.

[10] Chung KK, Thomas B, Li X, Pletnikova O, Troncoso JC (2004). S-nitrosylation of parkin regulates ubiquitination and compromises parkin's protective function.. Science.

[11] Jaffrey SR, Snyder SH (2001). The biotin switch method for the detection of S-nitrosylated proteins.. Sci STKE.

[12] Jakobs A, Koehnke J, Himstedt F, Funk M, Korn B (2007). Ubc9 fusion-directed SUMOylation (UFDS): a method to analyze function of protein SUMOylation.. Nat Methods.

[13] Hang J, Dasso M (2002). Association of the human SUMO-1 protease SENP2 with the nuclear pore.. J Biol Chem.

[14] Saitoh H, Pizzi MD, Wang J (2002). Perturbation of SUMOlation enzyme Ubc9 by distinct domain within nucleoporin RanBP2/Nup358.. J Biol Chem.

[15] Albor A, El-Hizawi S, Horn EJ, Laederich M, Frosk P (2006). The interaction of Piasy with Trim32, an E3-ubiquitin ligase mutated in limb-girdle muscular dystrophy type 2H, promotes Piasy degradation and regulates UVB-induced keratinocyte apoptosis through NFkappaB.. J Biol Chem.

[16] Depaux A, Regnier-Ricard F, Germani A, Varin-Blank N (2007). A crosstalk between hSiah2 and Pias E3-ligases modulates Pias-dependent activation.. Oncogene.

[17] Kudryashova E, Kudryashov D, Kramerova I, Spencer MJ (2005). Trim32 is a ubiquitin ligase mutated in limb girdle muscular dystrophy type 2H that binds to skeletal muscle myosin and ubiquitinates actin.. J Mol Biol.

[18] Uehara T, Nakamura T, Yao D, Shi ZQ, Gu Z (2006). S-nitrosylated protein-disulphide isomerase links protein misfolding to neurodegeneration.. Nature.

[19] Greco TM, Hodara R, Parastatidis I, Heijnen HF, Dennehy MK (2006). Identification of S-nitrosylation motifs by site-specific mapping of the S-nitrosocysteine proteome in human vascular smooth muscle cells.. Proc Natl Acad Sci U S A.

[20] Jones MC, Fusi L, Higham JH, bdel-Hafiz H, Horwitz KB (2006). Regulation of the SUMO pathway sensitizes differentiating human endometrial stromal cells to progesterone.. Proc Natl Acad Sci U S A.

[21] Jang HD, Yoon K, Shin YJ, Kim J, Lee SY (2004). PIAS3 suppresses NF-kappaB-mediated transcription by interacting with the p65/RelA subunit.. J Biol Chem.

[22] Chung CD, Liao J, Liu B, Rao X, Jay P (1997). Specific inhibition of Stat3 signal transduction by PIAS3.. Science.

[23] Tatham MH, Jaffray E, Vaughan OA, Desterro JM, Botting CH (2001). Polymeric chains of SUMO-2 and SUMO-3 are conjugated to protein substrates by SAE1/SAE2 and Ubc9.. J Biol Chem.

[24] Gostissa M, Hengstermann A, Fogal V, Sandy P, Schwarz SE (1999). Activation of p53 by conjugation to the ubiquitin-like protein SUMO-1.. EMBO J.

[25] Lyst MJ, Nan X, Stancheva I (2006). Regulation of MBD1-mediated transcriptional repression by SUMO and PIAS proteins.. EMBO J.

[26] Matsuura T, Shimono Y, Kawai K, Murakami H, Urano T (2005). PIAS proteins are involved in the SUMO-1 modification, intracellular translocation and transcriptional repressive activity of RET finger protein.. Exp Cell Res.

[27] Navarro-Lerida I, Corvi MM, Barrientos AA, Gavilanes F, Berthiaume LG (2004). Palmitoylation of inducible nitric-oxide synthase at Cys-3 is required for proper intracellular traffic and nitric oxide synthesis.. J Biol Chem.

[28] Man JH, Li HY, Zhang PJ, Zhou T, He K (2006). PIAS3 induction of PRB sumoylation represses PRB transactivation by destabilizing its retention in the nucleus.. Nucleic Acids Res.

[29] Liu GH, Qu J, Shen X (2006). Thioredoxin-mediated negative autoregulation of peroxisome proliferator-activated receptor alpha transcriptional activity.. Mol Biol Cell.

[30] Iwasaki K, Hailemariam K, Tsuji Y (2007). Protein inhibitor of activated STAT3 (PIAS3) interacts with activating transcription factor 1 (ATF1) and regulates the human ferritin H gene through an antioxidant responsive element.. J Biol Chem.

