# Supplementary figures and images for: Nitric Oxide Destabilizes Pias3 and Regulates Sumoylation
Source: PLoS One. 2007 Oct 31;2(10):e1085. doi: 10.1371/journal.pone.0001085 (PMC2064872; doi:10.1371/journal.pone.0001085)

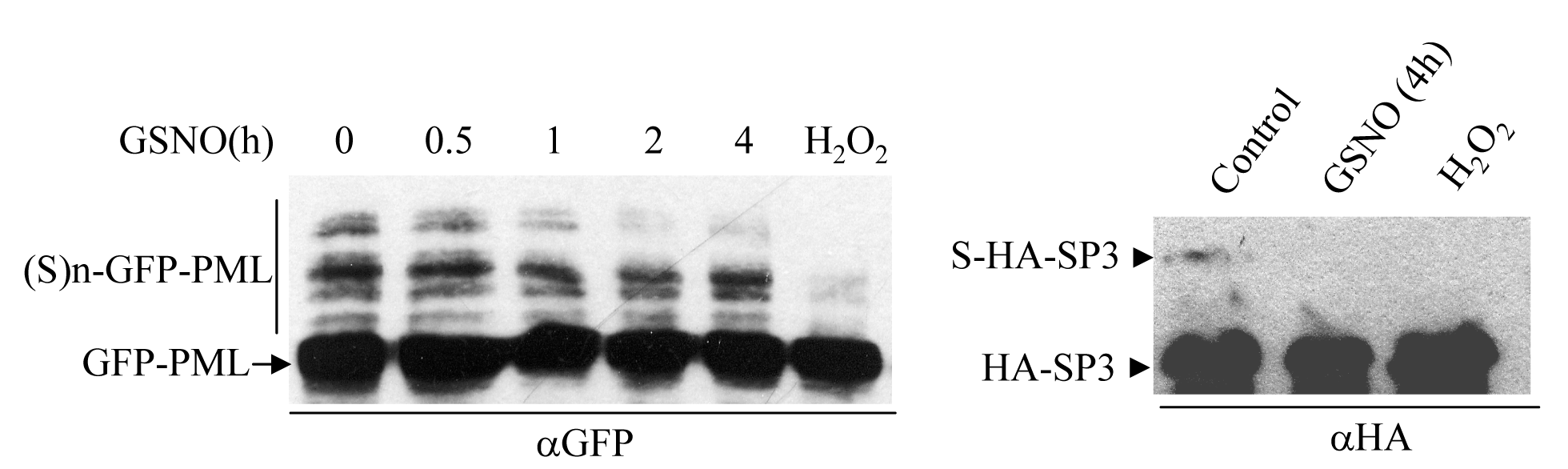

Supplement: Figure S1 — HEK293 cells transfected with GFP-PML (left) or HA-SP3 (right) were treated with H2O2 (1 mM) for 1h or with GSNO (0.5 mM) for indicated time, lysed in Laemmli buffer and immunoblotted with anti-GFP or anti-HA. (0.29 MB TIF) [file pone.0001085.s001.tif]

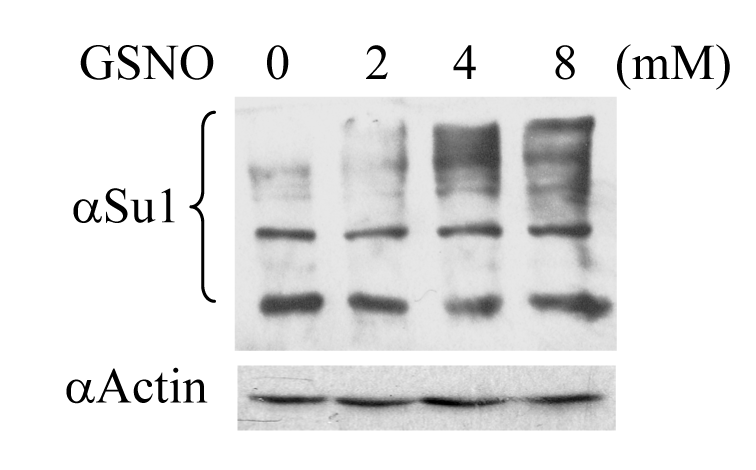

Supplement: Figure S2 — HeLa cells were treated with increasing concentrations of GSNO for 4 h, lysed in Laemmli buffer, and immunoblotted with anti-SUMO1 or anti-actin. (0.09 MB TIF) [file pone.0001085.s002.tif]

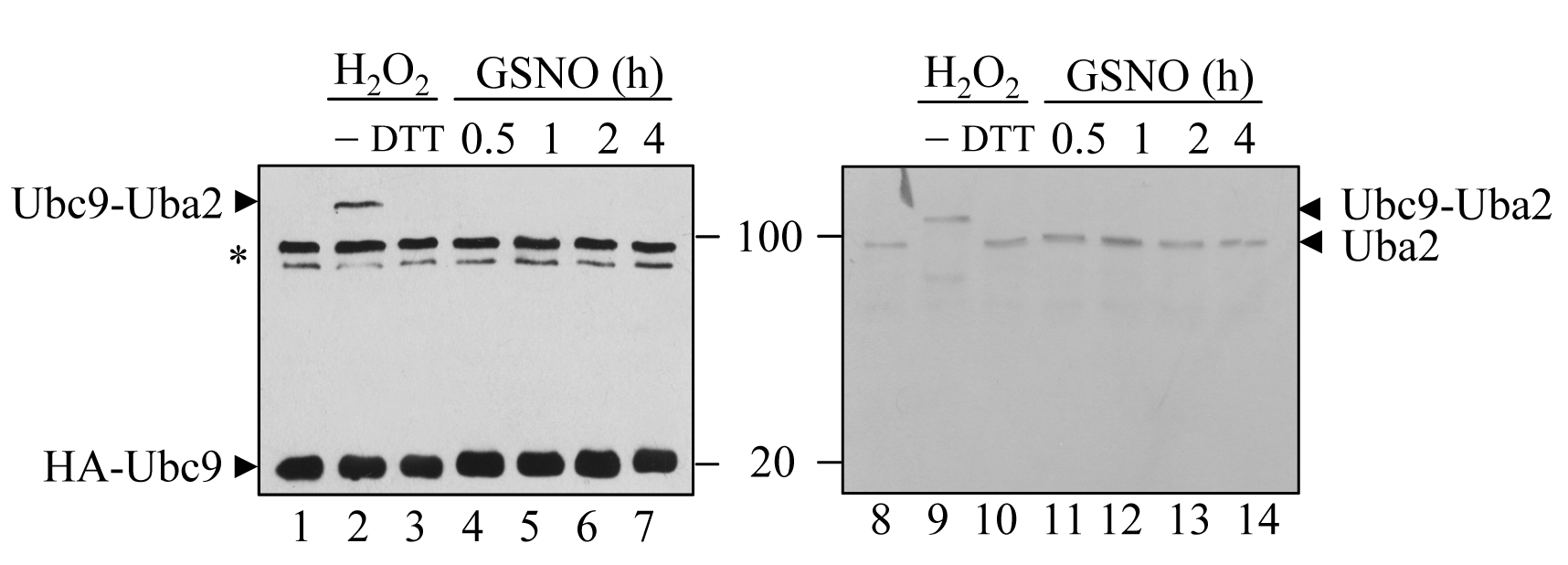

Supplement: Figure S3 — HeLa cells transfected with HA-Ubc9 were incubated with 0.5 mM H2O2 for 30 min or with 0.5 mM GSNO for the indicated time, lysed in Laemmli Buffer with (lanes 3 and 10) or without (other lanes) DTT and immunoblotted with either anti-HA (left) or anti-Uba2 (right). Asterisk: unspecific crossreacting band. (0.21 MB TIF) [file pone.0001085.s003.tif]

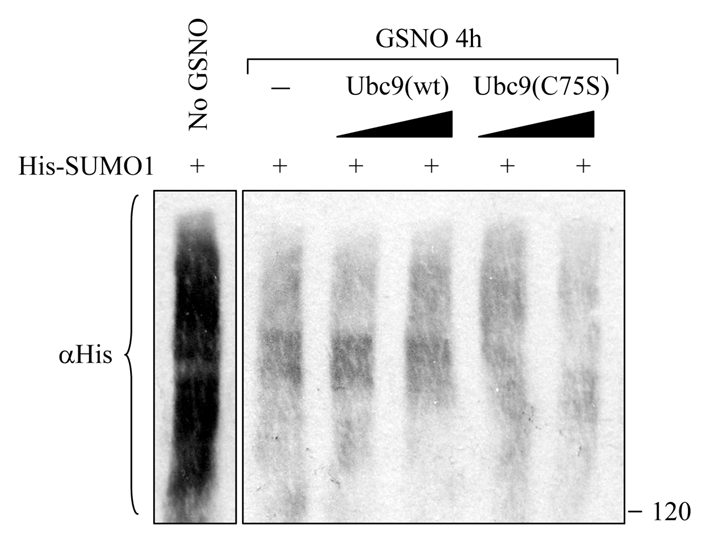

Supplement: Figure S4 — HeLa cells were cotransfected with His-SUMO1 and increasing amounts (from 30 to 300ng) of HA-Ubc9(WT) or HA-Ubc9(C75S), treated with 0.5 mM GSNO for 4 h, lysed in Laemmli buffer, and immunoblotted with anti-His. (0.39 MB TIF) [file pone.0001085.s004.tif]

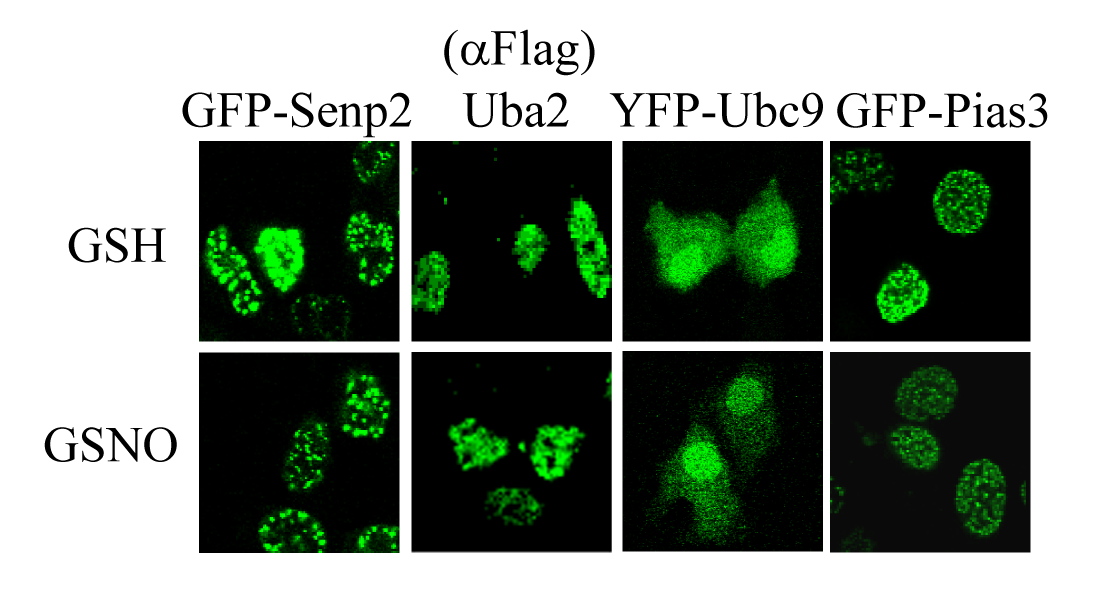

Supplement: Figure S5 — HEK293 cells were transfected with the indicated plasmids and subjected to immunofluorescent (for Flag-Uba2) or fluorescent protein imaging. (0.26 MB TIF) [file pone.0001085.s005.tif]

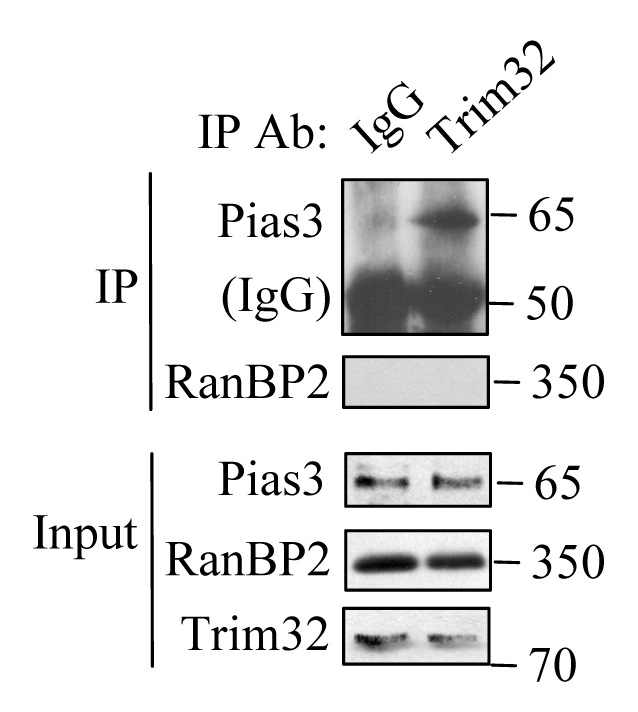

Supplement: Figure S6 — Co-IP analysis of the Pias3-Trim32 interaction in MG132-treated HeLa cells using anti-Trim32 (or control IgG) as the IP antibody. No coimmunoprecipitation was found between Trim32 and RanBP2, indicating a specific interaction between Trim32 and Pias3. (0.07 MB TIF) [file pone.0001085.s006.tif]

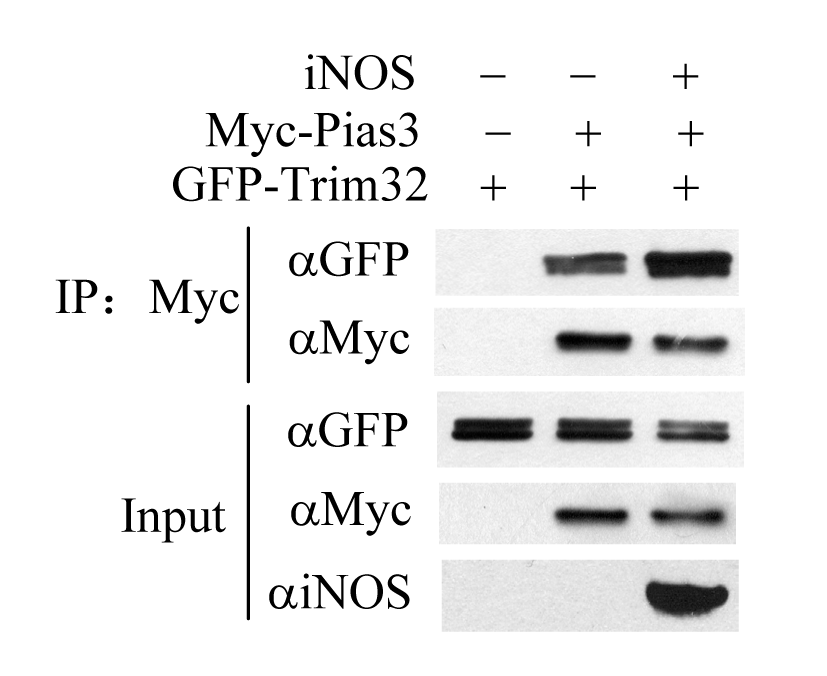

Supplement: Figure S7 — Co-IP analysis of Pias3-Trim32 interaction in transfected HEK293 cells. HEK293 cells were cotransfected with the indicated plasmids. 36h later, cells were treated with MG132 for 4h. The lysates were subjected to CO-IP assay using anti-Myc as the IP antibody. The expression of Myc-Pias3, GFP-Trim32 and/or iNOS in immunoprecipitates and cell lysates were monitored with the indicated antibodies. (0.11 MB TIF) [file pone.0001085.s007.tif]

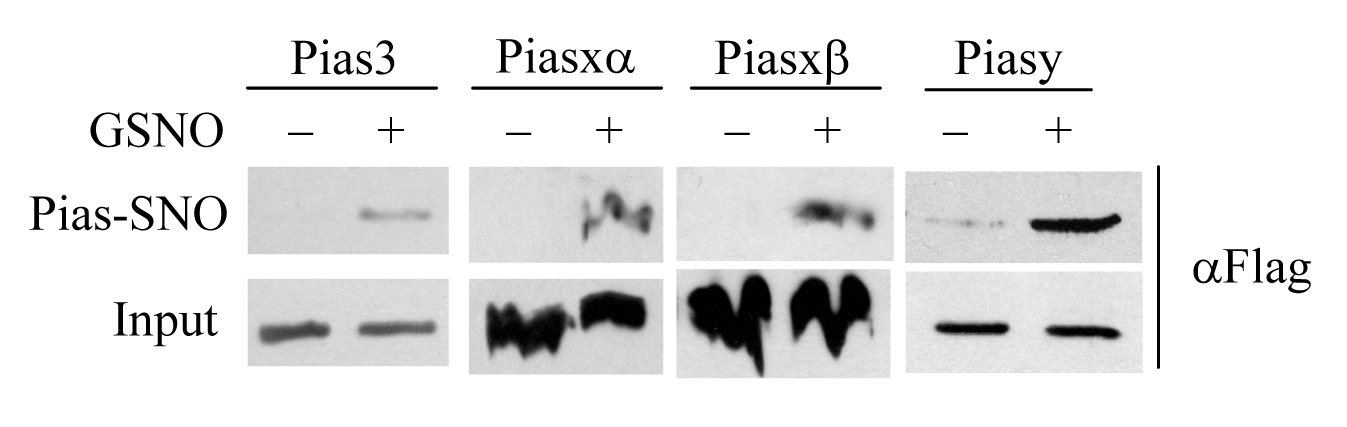

Supplement: Figure S8 — S-nitrosation of various Flag-tagged Pias subtypes expressed in HEK293 cells in the presence of 0.5 mM GSNO was determined by biotin-switch assay. (0.13 MB TIF) [file pone.0001085.s008.tif]

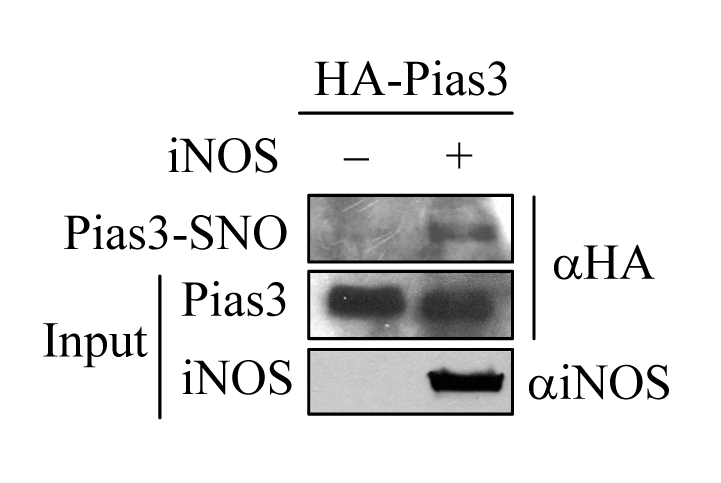

Supplement: Figure S9 — S-Nitrosation of HA-Pias3 in iNOS-overexpressed HEK293 cells. (0.05 MB TIF) [file pone.0001085.s009.tif]

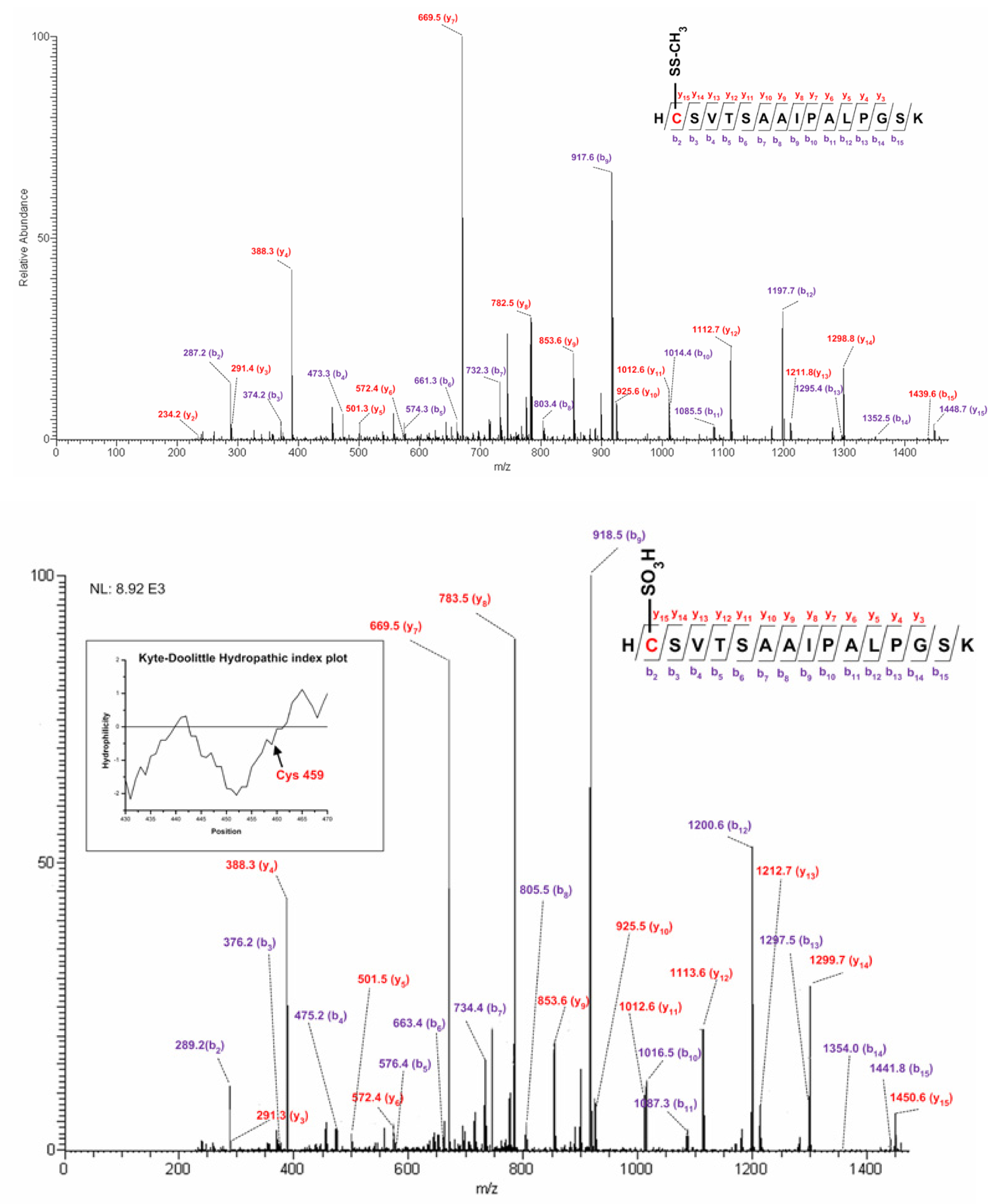

Supplement: Figure S10 — Nano-LC-MS/MS analysis of Pias3 before and after 0.1 mM GSNO treatment indicating cysteine-459 (C459) on peptide 458HCSVTSAAIPALPGSK473 was the modified site of S-nitrosation. The m/z 795.22+ precursor ion corresponds to the Pias3 peptide 458HCSVTSAAIPALPGSK473 with cysteine sulphonation in the GSNO-treated sample. Cysteine sulphonation was only observed after exposure to the NO donor GSNO. Analysis of the informative fragment ions y14+, y15+ confirmed the presence of the sulphonated cysteine. Kyte-Doolittle hydropathic index plot from the region flanking the identified S-nitrosocysteine residue (arrow) also showed that Cys459 was located within a hydrophobic pocket. The hydropathy plot was constructed by using a window of 13 amino acids. (1.73 MB TIF) [file pone.0001085.s010.tif]

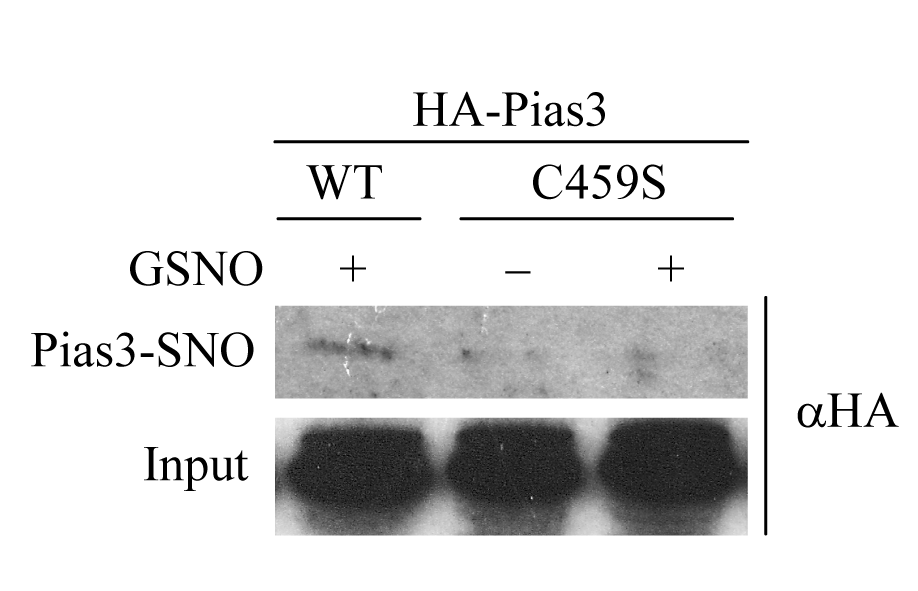

Supplement: Figure S11 — S-nitrosation of HA-Ubc9 or its C459S expressed in HEK293 cells in the presence of 0.5 mM GSNO was determined by biotin-switch assay. (0.11 MB TIF) [file pone.0001085.s011.tif]
